# NDV-induced apoptosis in absence of Bax; evidence of involvement of apoptotic proteins upstream of mitochondria

**DOI:** 10.1186/1743-422X-9-179

**Published:** 2012-08-30

**Authors:** Aidin Molouki, Khatijah Yusoff

**Affiliations:** 1 Institute of Biosciences, Universiti Putra Malaysia, 43400 UPM, Serdang, Selangor DE, Malaysia; 2 Department of Microbiology, Faculty of Biotechnology and Biomolecular Sciences, Universiti Putra Malaysia, 43400 UPM, Serdang, Selangor DE, Malaysia

## Abstract

**Background:**

Recently it was shown that following infection of HeLa cells with Newcastle disease virus (NDV), the matrix (M) protein binds to Bax and subsequently the intrinsic pathway of apoptosis is activated. Moreover, there was very little alteration on mRNA and protein levels of Bax and Bcl-2 after infection with NDV.

**Finding:**

In order to further investigate the role of members of the Bcl-2 family, Bax-knockout and wild-type HCT116 cells were infected with NDV strain AF2240. Although both cells underwent apoptosis through the activation of the intrinsic pathway and the release of cytochrome c from mitochondria, the percentage of dead Bax-knockout cells was significantly lower than wt cells (more than 10% at 48 h post-infection). In a parallel experiment, the effect of NDV on HT29 cells, that are originally Bcl-2-free, was studied. Apoptosis in HT29 cells was associated with Bax redistribution from cytoplasm to mitochondria, similar to that of HeLa and wt HCT116 cells.

**Conclusion:**

Although the presence of Bax during NDV-induced apoptosis contributes to a faster cell death, it was concluded that other apoptotic protein(s) upstream of mitochondria are also involved since cancer cells die whether in the presence or absence of Bax. Therefore, the classic Bax/Bcl-2 ratio may not be a major determinant in NDV-induced apoptosis.

## Findings

Newcastle disease virus (NDV) is a highly contagious avian virus that belongs to the genus *Avulavirus* from the family of *Paramyxoviridae*[1,2]. NDV is mostly recognized for its economic damage to the poultry industry worldwide since it causes high mortality rates in most species of bird [3,4]. NDV strains are mainly grouped into three major pathotypes on the basis of their pathogenicity [5,6], where lentogenic strains cause clinically low or unapparent respiratory disease, mesogenic strains cause intermediate symptoms and have moderate mortality rate, and lastly velogenic strains that cause severe intestinal lesions and neurological effects with high mortality rates. Malaysian NDV strain AF2240 is a viscerotropic velogenic strain that was isolated during an outbreak in the country in the 1960s and it is now mostly used as the challenge virus in vaccine trials in Malaysia [7]. On the other hand, NDV has also gained a lot of interest in cancer virotherapy since it can selectively kill human cancer cells [5]. It has been shown that NDV induces apoptosis in cancer cells by activating the mitochondrial pathway [8,9]. The proteins of the Bcl-2 family are known central regulators of this pathway [10]. Bcl-2 protein itself acts as pro-survival factor while pro-apoptotic members such as Bax initiate apoptosis [11]. All members of the Bcl-2 family interact with each other or other proteins via their conserved domains known as the Bcl-2 homology (BH) domains 1 to 4 [12]. Bcl-2, Bcl-XL, Bax, Bak, Bid and Bad are known members to regulate the mitochondrial membrane permeability transition pores (MPTP) [13-16] to act as checkpoints of apoptosis. We recently showed that the matrix (M) protein of NDV directly interacts with human Bax protein in HeLa cells through its BH3 domain [17]. This interaction activates Bax and subsequently initiates the release of cytochrome c from mitochondria to further activate the intrinsic pathway. Activation of some caspases such as caspase-3 in several cell lines following NDV infection has been frequently reported as well [8,18-20].

### Bax-knockout cells are more resistant to NDV infection

In the current study, the effect of NDV on cancer cells in the absence of Bcl-2 and Bax was investigated; HT29 human colon adenocarcinoma cell line (originally Bcl-2-free, purchased from ATCC), HCT116 Bax−/− (kindly provided by Bert Vogelstein, Johns Hopkins Medical University, MD, USA) and wt colorectal carcinoma cell lines (kindly provided by Eric Stanbridge, University of California, Irvine, CA, USA) were cultured in 6-well plates and infected with NDV strain AF2240 according to a standard protocol (30 HA units of NDV for 1.0 × 10^6^ cultured cells) [21]. Bax-deficient HCT116 cells were generated by targeted inactivation of the wild-type Bax allele in a Bax heterozygote [22].

**Figure 1:**
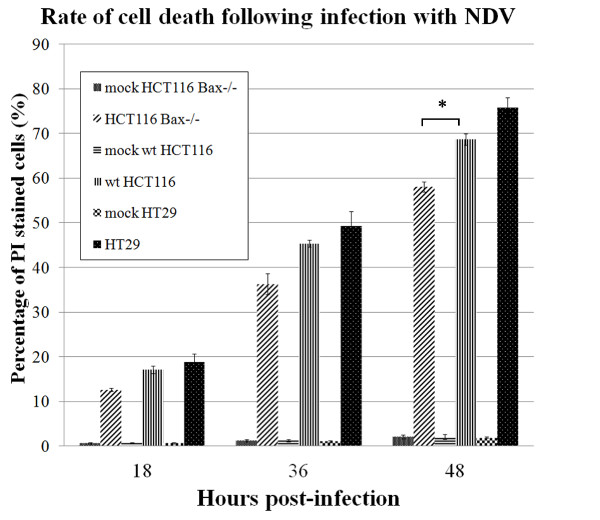
**Comparison of the percentage of PI stained NDV-infected cells.** Data are obtained at different time points 18 h, 36 h and 48 h by flowcytometry. Error bars indicate standard error of the mean from three independent measurements. The difference in the percentage of cell death in HCT116 Bax−/− cells compared to wt HCT116 at 48 h post-infection was statistically significant. *, p < 0.05.

Morphological changes and membrane blebbing was completely evident in these cell lines following infection. At different time points post-infection (18 h, 36 h and 48 h), the cells were checked by flowcytometry and PI staining for the number of dead cells, and graphs were drawn accordingly (Figure 1). At 48 h post-infection 75.76% of HT29 cells, 68.71% of wt HCT116 cells and only 58.09% of HCT116 Bax−/− cells were stained positive. The difference between the percentages of dead cells in HCT116 Bax−/− cell line compared to wt HCT116 was statistically significant.

### Translocation of Bax from cytoplasm to mitochondria

**Figure 2:**
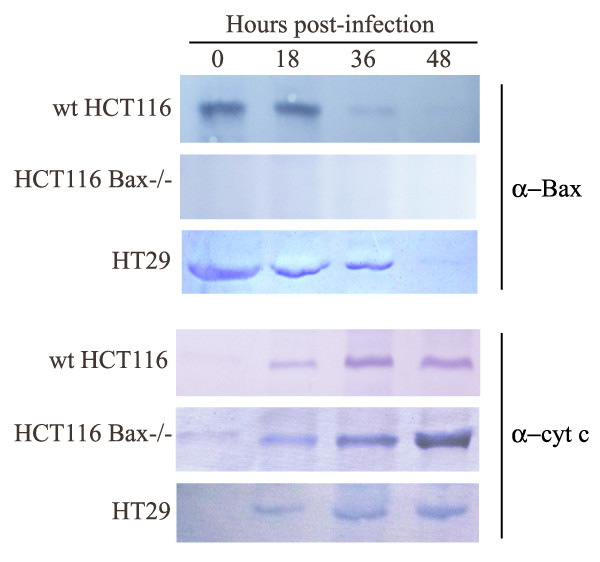
**NDV infection leads to translocation of Bax and release of cytochrome c into cytosol in colon cancer cells.** Infected wt HCT116, HCT116 Bax −/− and HT29 cells (18 h, 36 h and 48 h post-infection) were subjected to subcellular fractionation. The cytosolic samples were analyzed by Western blotting with anti-Bax clone 2D2 and anti-cytochrome c clone 7H8 antibodies. Absence of Bax in HCT116 Bax−/− cells was also checked.

Later, cytosolic and mitochondrial fractions of the NDV-infected cells were collected as previously described [9] and the samples were subjected to SDS-PAGE and Western blotting with anti-Bax clone 2D2 antibody (Zymed, USA) (Figure 2). HT29 and wt HCT116 cell lines showed that following NDV infection the amount of Bax increased in the mitochondrial fraction (not shown) while Bax became less detectable in the cytosolic fraction. Absence of Bax in knockout HCT116 cells was also checked.

### Activation of intrinsic pathway in NDV-infected Bax-knockout cells

Moreover, in order to investigate if the intrinsic pathway in the NDV-infected cells is activated, the ability of these cells to release cytochrome c from the mitochondria was also examined by subjecting the cytosolic fractions to Western blotting with anti-cytochrome c clone 7H8 antibody (Biovision, USA) (Figure 2). Similar to what was previously reported for HeLa cells [9], translocation of Bax from the cytosol into the mitochondria in NDV-infected HT29 and wt HCT116 cell lines was followed by the release of cytochrome c from mitochondria into the cytosol.

In addition, the amount of cytochrome c in the cytosolic fraction of the NDV-infected HCT116 Bax−/− cells also increased by time, suggesting that the mitochondrial pathway of apoptosis in this cell line is activated even in the absence of Bax.

## Discussion

In this study, after several trials, it was found that the percentage of cell death in NDV-infected Bax-knockout cells was lower compared to wt cells. This showed that the presence of Bax (and its subsequent interaction with the viral proteins such as the M protein) speeds up the apoptosis event. Since the Bax-knockout cells die following infection with the virus, it can be concluded that NDV is able to interact with apoptotic proteins other than Bax as well. The release of cytochrome c into cytosol proves that the mitochondrial pathway is activated through some other interaction(s) that directly or indirectly affected the proteins of the surface of mitochondria, allowing formation of pores that release mitochondrial factors.

Many Bcl-2 family members interact with each other via the BH domains [12]. For instance, Bax binds to several of these proteins by its BH3 domain [23,24] resulting in a series of complex interactions. Since the M protein of NDV contains a BH3 domain that interacts with Bax, it could be possible that in the absence of Bax, the M protein activates other pro-apoptotic members. However, so far this viral protein has shown no interaction with Bad, Bcl-XL and BimL [17]. Fusion (F) protein also failed to interact with the mentioned Bcl-2 members. Moreover, the BH3-like regions on the large (L) protein of NDV have not yet been examined for their ability to bind to Bcl-2 family proteins.

Bcl-2 normally blocks Bax oligomerization to inhibit its insertion into the mitochondria [10]. Since NDV infection also leads to apoptosis in absence of Bax, the classic Bax/Bcl-2 ratio [10-12] may not be a major determinant in NDV-induced apoptosis. This statement is in agreement with our previous findings that there was very little alteration on mRNA and protein levels of Bax and Bcl-2 following infection with NDV [9,17]. Moreover, little is known about the role of anti-apoptotic members of the Bcl-2 family during NDV infection. Since HT29 cells are Bcl-2-free, it will be interesting to check if the absence of Bcl-2 has a significant role in NDV-induced apoptosis at all, or its overexpression has any effect on the apoptosis rate.

Bak and Bax have almost the same protein structure, share similar BH domains and are the only two pro-apoptotic Bcl-2 family proteins discovered so far that form oligomeric pores on the mitochondria surface. Bak can independently release cytochrome c from mitochondria in absence of Bax [25], and therefore these characteristics make it an excellent candidate for future studies. Although Bax is a cytosolic protein and Bak localizes to mitochondria, they both facilitate the release of cytochrome c from mitochondria through MPTP pores [13,26].

BH3-only members such as Bid and Bad are located upstream of mitochondria that upon activation move towards mitochondria to, respectively, activate Bak and Bax [16] and inhibit Bcl-2 and Bcl-XL function [15] to facilitate release of mitochondrial factors. Therefore, if NDV proteins directly or indirectly (through activation of BH3-only protein) activate Bak in HCT116 Bax−/− cells then perhaps this could be a possible reason behind the release of cytochrome c. Use of Bak−/− cells in future studies will definitely let us investigate this hypothesis further. On the other hand, it has been previously shown that NDV kills Bcl-XL-overexpressing cells that are resistant to Bak overexpression [27] and also overcomes the anti-apoptotic function of Livin [28], a novel member of the inhibitor of apoptosis protein (IAP) family frequently overexpressed in melanoma. Moreover, it has been found that during infection with RNA viruses a BH3-like domain of IRF-3 mediates binding to cytosolic Bax (but not Bcl-2, Bcl-XL or Bak) to induce Bax activation, culminating in cytochrome c release and apoptosome formation [29]. Together it could be further suggested that proteins located upstream of mitochondria are strong candidates for such activities.

Overall, it was concluded that Bax is not the only cellular protein that NDV proteins interact with to trigger apoptosis. However, Bax plays a role in the apoptosis of HCT116 cells, since the lack of Bax contributes to slower cell death. This further suggested that the interaction between Bax and NDV proteins, such as that with the M protein [17], is crucial and acts as determinant during NDV-induced apoptosis. Further study is needed to find other possible interactions between NDV proteins and cellular apoptotic proteins.

## Abbreviations

ATCC: American type culture collection; BH domain: Bcl-2 homology domain; wt: Wild-type.

## Competing interests

The authors declare that they have no competing interests.

## Authors’ contributions

AM designed and performed the experiments and drafted the manuscript. KY participated in the design of the study, financial support and contributed to writing the manuscript. Both authors have read and approved the final manuscript.
